# Supplementary material for: Working with what you have: How the East Africa Preterm Birth Initiative used gestational age data from facility maternity registers
Source: PLoS One. 2020 Aug 31;15(8):e0237656. doi: 10.1371/journal.pone.0237656 (PMC7458293; doi:10.1371/journal.pone.0237656)
Supplement: Attachment — Submitted filename: Reviewers_Comments_Miller_PLOSOne.docx [file pone.0237656.s002.docx]

**Working with what you have: how the East Africa Preterm Birth Initiative adjusted to limited gestational age data in routinely collected birth registers**

Date: 09 June 2020

Thank you for the opportunity to review this manuscript by Miller and colleagues. Generally this is a well written paper that clearly articulates the challenges of accurate preterm birth ascertainment in low resourced settings without access to early antenatal ultrasound and evaluates a pragmatic approach to improving the accuracy of preterm birth outcome classification through an implementation science study. Although the approach is still imperfect, the manuscript will be helpful to others working with similar quality gestational age information to understand the limitations.

Major comments:

- Data strengthening occurred in April in Uganda and in June in Kenya, with data collection spanning this period from March-September 2016.
  - Was any impact seen on the quality of the data before and after the data strengthening in each country? This would be quite helpful to understand whether investment in training to improve accuracy is effective and worthwhile.
  - Considering large inter-country variation in gestational age estimation accuracy and ineligible births, were there any differences in the format, content or timing of training in the 2 different countries that can be learnt from to improve GA accuracy.
- Line 131 – To consider that term infants with fetal growth restriction could also be <2500g at birth. This might be particularly relevant in countries with high prevalence of smoking and alcohol in pregnancy that contribute to fetal growth restriction. In these countries preterm birth could be over-estimated using the PTBi-EA criteria.
- Regarding ineligible births (reflected in Table 1) - Are there differences by country in considering <500g as miscarriages and not recording in them in the maternity register? If a country more often includes miscarriages in the maternity register then this could account for the higher proportion of “ineligible births”.

Minor comments and corrections:

- Abstract Objective 2^nd^ sentence – think it should reach “chose an eligibility criterion (singular) that…” or otherwise drop the an “chose eligibility criteria that…”
- Line 110 – might be clearer to the reader to specify that the intrapartum and immediate postnatal quality of care improvement package was specifically for women in preterm labour (if this is correct).
- Line 176 – include citations
