# Supplementary material for: Working with what you have: How the East Africa Preterm Birth Initiative used gestational age data from facility maternity registers
Source: PLoS One. 2020 Aug 31;15(8):e0237656. doi: 10.1371/journal.pone.0237656 (PMC7458293; doi:10.1371/journal.pone.0237656)
Supplement: Attachment — Submitted filename: Comments to Authors.docx [file pone.0237656.s003.docx]

**Comments to Authors**

The authors describe the results of a retrospective analysis evaluating the quality of gestational age data in maternity registers from study facilities in Kenya and Uganda. Estimation of preterm birth burden at population level is known to be complicated by data quality and availability, as such, this manuscript addresses an important public health concern.

In this manuscript, the authors report concerns about the quality of gestational age data in maternity registers; and present practical inclusion criteria for identifying preterm infants using routinely collected data when there are questions about GA data quality.

I have the following major and minor comments:

Introduction

1. This section could perhaps be strengthened by highlighting the following
   - central importance of gestation age and its correlation with developmental processes
   - inherent imprecision of gestational given its control by multiple physiological processes (natural variability and/or estimation error)

Methods

1. *“Methods and procedures are described in detail elsewhere”*
   - While the authors direct readers to the published study protocol for further detail, there are details of the procedures of the primary study that are central to this analysis that could be included or presented clearer to contextualize this analysis e.g. Intervention delivery at facility level and outcome assessment at facility and individual level could be useful information as well as the primary outcome of the primary study
2. Could the authors provide more information about the maternity registers
   - Are these completed at delivery? If completed at delivery what is the source of the data – is this based on self-report or patient-held records completed throughout the antenatal period?
     - gestational age data: is this calculated by the healthcare workers, based on gestational age assessment completed at the first antenatal visit? Or is it based on an assessment done when women present at the facilities during early stages of labour? is there any indication whether GA recorded is based on antenatal or postnatal assessment?
     - Is there any information of timing of presentation for antenatal booking? JT Price et al showed the importance of this information through their observation that bias in LMP based GA estimates increased as the gestation at antenatal care presentation advanced.
3. *“initial data strengthening in April (Uganda) and June (Kenya) 2016 which included a review of GA assessment methods….”* and “….*extraction of the maternity register data from each of the 23 facilities from* ***March - September 2016****”*
   - Could the authors indicate that this activity was part of the intervention packages of the primary study in order to “strengthen existing data collection processes in health facilities, introducing standard tools to improve GA assessment..”
   - Since this data strengthening took place during data collection (particularly in Kenya) was the data completeness, consistency and plausibility – assessed before and after intervention ?
   - Given the differences in timing of intervention – if differences were seen before and after the intervention perhaps this could be touched upon in the limitation section
4. *“Completeness was calculated as a proportion of all births where GA, birth weight, sex, and birth outcomes were recorded”*
   - Could the authors clarify that completeness was assessed for each of these variables separately
5. *“consistency evaluation was conducted only for Kenyan data as their maternity registers list both GA and LMP”*
   - Presumably the LMP is the date? Could the authors make this clearer
   - Is it known what the ‘recorded GA’ was based on? Is there information on method of assessment use?
   - Was there any GA assessment based on symphysis fundal height?

Results

1. Could the authors consider describing the ineligible births further in the results section
   - which exclusion criteria had the highest proportion of ineligible births? Were there any differences noted by country?
2. *“The calculated GAs ranged from -14.3 to 91.9 weeks…”*
   - Were any of these LMPs excluded based on implausibility or were all included regardless of plausibility?
3. Is there any data on mode of delivery? – to enable distinction between provider initiated and spontaneous preterm births
4. *“initial data strengthening in* ***April (Uganda) and June (Kenya)*** *2016 which included a review of GA assessment methods….”*
   - Were there improvements noted after the data strengthening intervention?

Discussion

1. Highlighting the background rates of preterm births in Kenya and Uganda earlier in this section before the different inclusion criteria approaches are being discussed would be useful
   - Besides estimates presented by Chawanpaiboon et al are there any other preterm birth estimates reported from national statistics or from studies in the countries that could also be discussed in relation to estimates observed in this study?
2. Could the authors consider highlighting the following points
   - the difficulty of preterm birth prevention given that it is a complex syndrome with interrelated aetiological factors and pathways contributing to phenotypical differences, and thus requiring a suite of evidence-based interventions
   - gestation based definitions of preterm birth are the most commonly used as they are better short- and long-term outcome predictors than measures of intrauterine growth. This could be followed by a discussion of the reasons why using gestation based definitions is not always possible and the other methods available
3. *“LBW definition…is a natural proxy for preterm birth when GA data are poor”*
   - Important to highlight that using LBW is a postnatal proxy which is useful for identifying preterm births in particular instances such as for clinical management as well as screening for additional postnatal clinical surveillance, interventions or trials such as the PTBi-EA.
   - While the authors mention that using birthweight may “*miss late preterm infants that are not LBW”* – perhaps a stronger point could be made that in instances where the goal is to accurately determine preterm birth this proxy measure maybe less useful because while approximately 2/3 of LBW infants are due to preterm birth, a proportion are term infants who are growth restricted
4. Perhaps the authors could expand on *“this pragmatic approach to screening allows research to be conducted without substantial investment of early pregnancy ultrasound”* and underscore in the conclusion the need to invest in improving GA assessment methods during antenatal care and improving data quality of routinely collected data at population levels inorder to improve identification of preterm infants
5. *“…this is not surprising given that the PTBi-EA study sites were government hospitals In rural, malaria endemic areas”*
   - Could the authors expand on this point and discuss the discrepancy with Chawanpaiboon et al rates in more detail
6. *“…in preterm birth estimates over 80% of stillbirths are also preterm ….”*
   - How were stillbirths classified? Were they all classified as preterm births
7. Limitation section could be expanded to include a discussion of the methodological challenges of using routinely collected data for birth outcomes and how they relate specifically to PTBi-EA where enrolment occurs at delivery
8. Interpretation of these findings could also be strengthen by
   - discussion of the potential effect of the data strengthening intervention – and whether it had any impact on the GA estimates or recording and how this exercise/intervention can be used to strengthen future studies using routinely collected data
   - discussion about potential reasons for the differences in data quality between the two countries such as reasons for Uganda’s completeness plausibility

Minor comments

1. Evaluation of the “validity of the PTBi-EA eligibility criteria” is mentioned in the abstract as part of the aims of this analysis but not in the introduction
2. Line 111 – “*Methods and procedures are described in detail elsewhere*.” – check the reference
3. Line 176 “(CITE IG and protocol paper) – correct this
